# Bio and Synthetic Based Polymer Composite Materials

**DOI:** 10.3390/polym14183778

**Published:** 2022-09-09

**Authors:** R. A. Ilyas, S. M. Sapuan, Emin Bayraktar

**Affiliations:** 1 School of Chemical and Energy Engineering, Faculty of Engineering, Universiti Teknologi Malaysia (UTM), Johor Bahru 81310, Malaysia; 2 Centre for Advanced Composite Materials (CACM), Universiti Teknologi Malaysia (UTM), Johor Bahru 81310, Malaysia; 3 Institute of Tropical Forest and Forest Products (INTROP), Universiti Putra Malaysia, Serdang 43400, Malaysia; 4 Advanced Engineering Materials and Composites, Department of Mechanical and Manufacturing Engineering, Faculty of Engineering, Universiti Putra Malaysia, Serdang 43400, Malaysia; 5 School of Mechanical and Manufacturing Engineering, ISAE-SUPMECA Institute of Mechanics of Paris, 93400 Saint-Ouen, France

*Bio and Synthetic Based Polymer Composite Materials* is a newly opened Special Issue of *Polymers*, which aims to publish original and review papers on new scientific and applied research and make contributions to the findings and understanding of the reinforcing effects of various bio and synthetic-based polymers on the performance of polymer composites. This Special Issue also covers the fibre-reinforced polymer composites’ fundamentals, characterisation, and applications.

It is recognised that synthetic-based polymer or petroleum-based plastics have great barrier and thermomechanical properties, as well as a low production cost and require lightweight materials, which produce good performance in terms of overall criteria [1]. The environmental impact of petroleum-based plastic materials, which are non-biodegradable and the increasing need for more sustainable green materials, especially for packaging and plastics in particular, have become an issue of concern. These phenomena are ever-growing global concerns. Thus, in order to overcome this problem, solutions to reduce and in some cases to replace those petroleum-based plastic materials are prioritised in research efforts. One of the current focuses is replacing synthetic-based polymer with bio-based polymer, also known as biopolymers. In recent years, the development of biopolymers based on constituents obtained from natural resources has gained much attention [2,3]. The exploitation of biopolymers to engineer advanced biocomposites and hybrid composite materials is the focus of increasing scientific activity, explained by the growing environmental concerns and interest in the novel features and multiple functionalities of these macromolecules. Biopolymers such as thermoplastic starch (TPS), chitosan, polyhydroxyalkanoates (PHA), cellulose, lignin, chitin, polyhydroxybutyrate (PHB), and poly lactic acid (PLA) have been pursued as alternative solutions. The most widely used is PLA, which is mainly used in packaging applications. It is used for films or thermoformed or injected packages for relative short-term and mild temperature contact conditions, such as fresh salads and beverage drinks, because of its low resistance to temperature. One major limitation commonly referred to is its high price and commercial shortage, as compared to conventional plastics. Thermoplastic starch (TPS) has also been used for replacing petroleum-based plastics [4,5]. However, TPS has lower mechanical properties which make it unsuitable to be used in packaging applications [6]. Thus, one of the ways to overcome this problem is by reinforcing TPS with fiber, which can improve its mechanical properties.

On the other hand, synthetic fibers have been the leading commodity in the composites industry. However, synthetic fibers possess many disadvantages as they are non-biodegradable. Since synthetic fibers have many shortcomings, researchers have expressed growing interest in producing polymers that incorporate natural fibers [7,8,9]. Natural fibers are becoming more common as a viable option due to the harmful environmental and health consequences of synthetic fibers [10]. Concerns about the environment, the rising greenhouse effect and increasing interest in the use of sustainable materials has motivated researchers to investigate biocomposite materials. Today, fibre-reinforced polymers are used in several applications including in packaging [3,11,12]; electrical and electronic appliances [13,14]; crossarm structures [15]; foam structures [16]; as energy storage [17,18]; in automotives [19]; in filter, coating, and bone tissue engineering; in drug delivery [20]; human prosthetics [21]; and more. The continuous development and appearance on the market of new high-performance reinforcing fibers in polymer composites have constituted a strong challenge for researchers to design and adapt new functional composites for several applications [22,23,24,25,26,27,28]. Such natural fibers are comprised of various lignocellulosic plant fiber, cellulose, nanocrystalline cellulose [29,30,31,32], nanofibrillated cellulose [33,34], bacterial nanocellulose [35,36], and lignin nanoparticles [37]. The great interest in natural fiber composites is due to their high performance, biodegradability, non-abrasiveness, light weight, and low cost. Moreover, the widespread adoption of natural fibers and biopolymers as green materials is motivated by the rapid depletion of petroleum supplies, as well as by a growing recognition of global environmental issues associated with the use of traditional plastics. The successful application of biopolymers and the promise of alternative pathways with a reduced carbon footprint arising from the use of bio-based materials bodes well for the future design and development of ever more sophisticated green materials.

Thus, in this Special Issue, we aim to capture the cutting edge of the state of the art in research pertaining to bio and synthetic-based polymer composite materials and their advanced applications. Contributions to the processing of bio and synthetic polymers, the use of diverse polymer sources, the reinforcement of fiber materials with polymers, and applications of these polymers composites constitute the backbone of this Special Issue.
